# Design and Fabrication of Single-Walled Carbon Nanonet Flexible Strain Sensors

**DOI:** 10.3390/s120303269

**Published:** 2012-03-07

**Authors:** Ya-Ting Huang, Shyh-Chour Huang, Chih-Chao Hsu, Ru-Min Chao, Trung Kien Vu

**Affiliations:** 1 Department of Mechanical Engineering, National Kaohsiung University of Applied Sciences, Kaohsiung 80778, Taiwan; E-Mails: yating3133@yahoo.com.tw (Y.-T.H.); kienchaoban@yahoo.com (T.K.V.); 2 Systems and Naval Mechatronics Engineering, National Cheng-Kung University, Tainan 701, Taiwan; E-Mails: p1894101@mail.ncku.edu.tw (C.-C.H.); rmchao@mail.ncku.edu.tw (R.-M.C.)

**Keywords:** Parylene-C, single-walled carbon nanonets, strain sensor

## Abstract

This study presents a novel flexible strain sensor for real-time strain sensing. The material for strain sensing is single-walled carbon nanonets, grown using the alcohol catalytic chemical vapor deposition method, that were encapsulated between two layers of Parylene-C, with a polyimide layer as the sensing surface. All of the micro-fabrication was compatible with the standard IC process. Experimental results indicated that the gauge factor of the proposed strain sensor was larger than 4.5, approximately 2.0 times greater than those of commercial gauges. The results also demonstrated that the gauge factor is small when the growth time of SWCNNs is lengthier, and the gauge factor is large when the line width of the serpentine pattern of SWCNNs is small.

## 1. Introduction

Bone strains are vital factors for osteogenic responses [1]. A complete understanding of the biomechanical behavior of human bone under daily activity conditions is crucial for the development of post-surgical therapies, which must be adapted to the healing stage [2]. Real-time monitoring of the musculoskeletal condition through bone strain recording would help doctors gain instant and detailed information for rehabilitation monitoring, as well as improve clinical studies for advanced orthopedic operations [3]. *In vivo* bone strain measurement through an implantable sensor is one of the most suitable solutions for these requirements [4]. A high-sensitivity implantable monitoring sensor is required for measuring strain because strain is an easy and quick way to determine surface variation of the bone or the amount of joint compression. Metallic foil gauges are commercially available because of their low cost and stable fabrication, but they have some disadvantages, such as low sensitivity, large size, and low biocompatibility, which limit their application for long-term solutions. The large pattern of metallic foil gauges also presents difficulty in implantable surgery and causes patient discomfort.

Because of their reversible electromechanical [5,6] and biocompatible [7] characteristics, carbon nanotubes (CNTs) are excellent for bone strain gauges, in which the strain would be measured by recognizing electrical resistance changes [8–15]. CNTs are nanoscale materials that have interesting electrochemical, mechanical, and biological properties. They are macromolecules of carbon, and their elastic modulus is 1 TPa [16]. Two types of CNTs are available, that is, single-walled carbon nanotubes (SWCNTs) and multi-walled carbon nanotubes (MWCNTs). CNTs are assumed to be biocompatible with osteoblast cells, which have piezoresistive effect (the theoretical ability of the material for strain sensing) of up to 1,000, and a diameter of approximately 1 nm [17,18]. CNTs are fabricated using laser ablation, arc-discharge, and catalytic chemical vapor deposition (CCVD). However, laser ablation and arc-discharge do not result in mass growth of CNTs, and cannot be integrated into the MEMS process. However, CCVD can achieve mass growth of CNTs, and also allows sensors to increase the opportunity for major commercial productions. CNTs become carbon nanonets with a certain thickness by using CCVD. Thereby, the presented sensor was designed and fabricated with piezoresistive effect and MEMS process.

The implantable sensor requires three parts, namely substrate, piezoresistive material, and a waterproof layer. Parylene-C is a biocompatible flexible, and waterproof polymer, that is widely used for producing a variety of products, such as micropumps, micronozzles, neural probes, and cell manipulation platforms. The Young modulus of Parylene is between 2 and 5 GPa, which is lower than those of the human cortical bone (20.5 GPa) and trabecular bone tissue (18 GPa). Therefore, Parylene can be fully attached to the bone [19–21].

In this study, a flexible strain sensor was designed and fabricated based on the piezoresistive effect of single-walled carbon nanonets (SWCNNs), and Parylene-C was used as the waterproof layer for ensuring biocompatibility.

## 2. Sensor Design and Fabrication

### 2.1. Design

**Figure 1.**
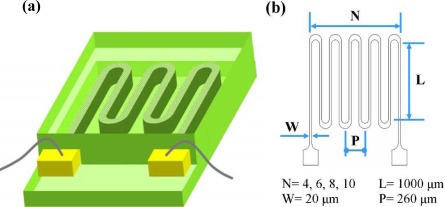
Strain sensor design: (**a**) 3D diagram (**b**) dimension of SWCNNs pattern.

The commercial strain sensors were assembled by wire, metal foil (sensing element), laminate film (waterproof layer), and a base substrate. The resistance of the metal foil gauge changes with strain during stress loading, and the strain can be determined by the change in electrical resistance. A serpentine pattern is commonly used for commercial strain sensors. In this study, we used single-walled carbon nanonets (SWCNNs) to replace the customary metal foil. Parylene-C was used as the waterproof layer, and another layer of Parylene-C was used as the base (Figure 1(a)). Further, to determine the Gauge Factor scale-down effects for the CNNs under different geometries, a serpentine pattern with different numbers of turns, a constant length and various widths of CNN was designed (Figure 1(b)).

### 2.2. Fabrication Process

**Figure 2.**
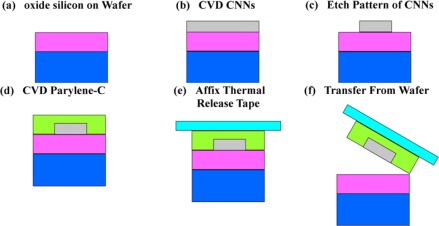

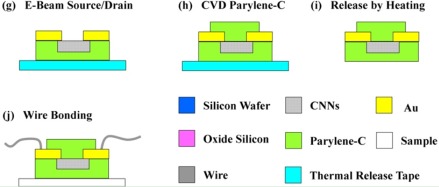
Fabrication process.

The manufacturing process for the presented strain sensing device is summarized in Figure 2. First, a 500 nm oxide layer is grown on a silicon wafer (Figure 2(a)). The surface of the SiO_2_ layer is not naturally hydrophilic. In order to make the surface hydrophilic for uniform coating with nanosize metal catalysts, the surface of the oxide substrate is first treated with standard cleaning solutions 1 and 2 (SC1 and SC2) then, subsequently dipped in a Co/Mo solution. After dipping, the wafer was placed into the alcohol catalytic chemical vapor deposition (ACCVD) apparatus for growing CNNs (Figure 2(b)). Next, Reactive Ion Etching was used to etch CNNs to form the serpentine pattern (Figure 2(c)). The wafer with the SWCNN pattern was subsequently placed into the Parylene deposition equipment (Specialty Coating System, Model PDS-2010) for a 6 μm thick Parylene-C coating. A thermal release tape (green layer) was used to bond the Parylene-C layer with the CNN serpentine pattern (Figure 2(d,e)).

Then, a gold layer is used as the source drain electrodes using the lift-off process (Figure 2(g)). Finally, the device was placed into the Parylene deposition equipment again for another Parylene-C coating to form the waterproof layer. After these fabrication steps, the presented strain sensor was released from the thermal release tape by heating for 1 min at 120 °C.

### 2.3. Single-Walled Carbon Nanonets Growth

**Figure 3.**
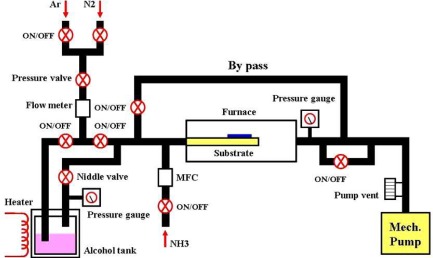
ACCVD equipment diagram [22].

**Figure 4.**
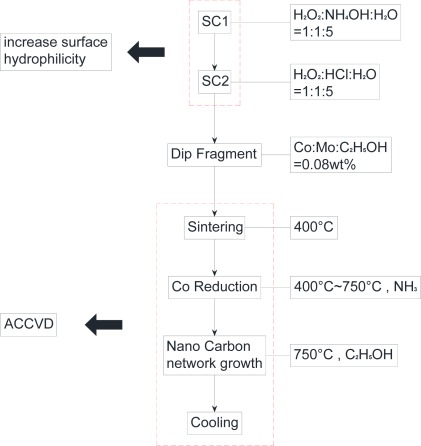
Single-walled carbon nanonets growth process flowchart.

In this study, the sensing material of the sensor, that is, single-walled carbon nanonets, which were grown using alcohol catalytic chemical vapor deposition equipment (ACCVD, as shown in Figure 3), was provided by National Nano Device Laboratories (NDL, Taiwan). The entire process is shown in Figure 4.

The process used dip set (Dip) to attach the catalytic metal to the fragments. The amount of attached catalytic metal attached is crucial; therefore, the SC1 and SC2 solutions were boiled to temporarily increase the surface hydrophilicity. The SC1 solution was prepared with H_2_O_2_-NH_4_OH-H_2_O in the ratio of 1:1:5, heated to 75 °C for 20 min. Subsequently, H2O_2_-HCl-H_2_O in the ratio of 1:1:6 was used to prepare the SC2 solution, which was heated to 75 °C for another 20 min. Then, the device was cleaned with deionized water and dipped in catalytic metal-Co/Mo solution, the ratio of which was 0.08 wt.% (Co: Mo-C_2_H_5_OH), for 10 min. Subsequently, the ACCVD was heated to 400 °C, and the stage quartz fragments were placed and sintered into ACCVD for 7 min and 30 s to allow the catalytic metal to be fixed on the Si substrate. The temperature in the ACCVD process was set to 750 °C, which resulted in gas formation, to restore the oxidation of the catalytic metal. The ACCVD was maintained at 750 °C environment during the growth stage of single-walled carbon nanonets for carbon—alcohol vapor in the chamber. Finally, only Ar gas was passed into the chamber for cooling to complete the growth of the single-walled carbon nanonets.

### 2.4. Fabrication Results

**Figure 5.**
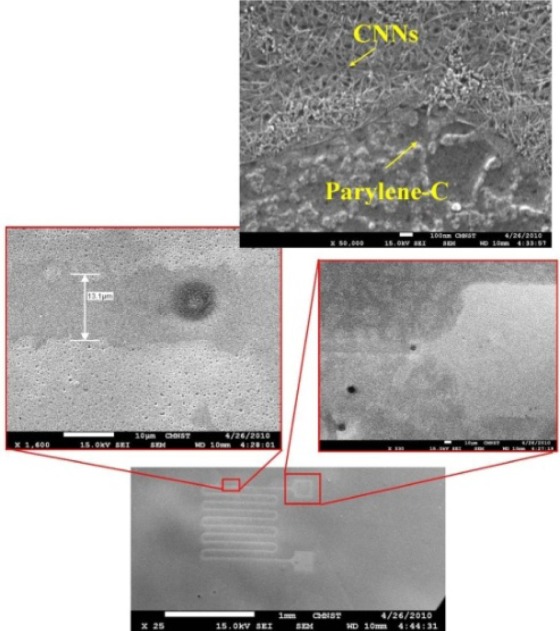
Nanocarbon network and SEM images of Parylene-C.

The inset in Figure 5 shows a closer view of the CNNs and Parylene-C, another closer view shows the transfer result with the 10 μm pattern after the Parylene-C is pulled off the silicon substrate.

**Figure 6.**
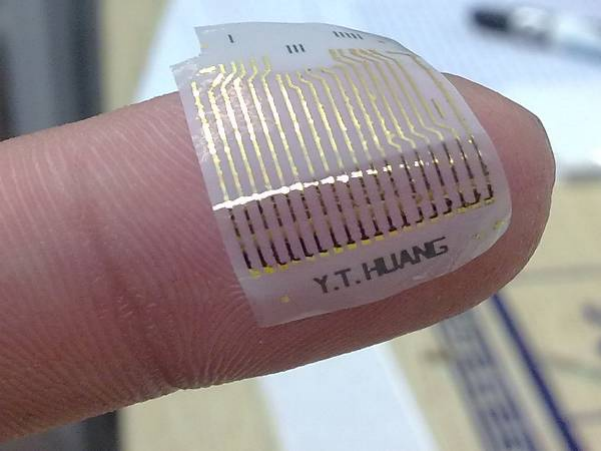
Flexible nanocarbon network strain sensor complete map.

In addition, the wider view of Figure 5 shows the CNNs serpentine pattern. Further, after the micro-fabrication, the device with thermal release tape is placed on the hot plate, and the presented strain sensor is released from the thermal release tape by heating for 1 min at 120 °C. The final flexible nano-carbon network strain sensor is shown in Figure 6.

## 3. Electromechanical Testing

An acrylic plate, of 200 mm length with a width of 50 mm, was placed in an Instron testing machine (model 1130). The Parylene-based SWCNNs strain sensor was attached on the plate with special glue “M-Bond 200” for the device at the length of 10 cm and the rear of the plate.

**Figure 7.**
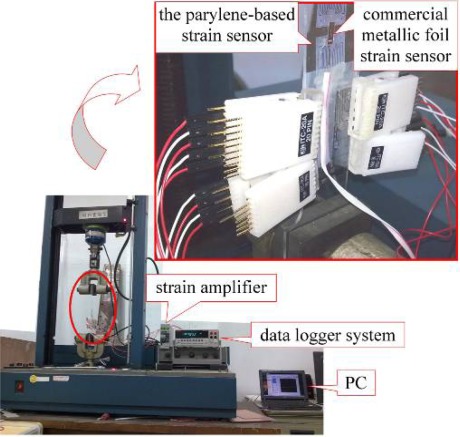
The photo of the tensile testing system.

The plate was then attached to a commercial metallic foil strain sensor (KYOWA Electronic Instruments Co., LTD). A strain amplifier (KYOWA, model DPM-711B) was used to obtain the output strain signal as voltage (1 V = 5,000 micro-strain) from the commercial metallic foil strain sensor (The voltage input for the metal foil gauge is 3.7 V). The output signal of resistance from the Parylene-based SWCNNs strain sensor was acquired by a data logger system (Keithley, model 2700), the strain of the plate was measured by a 1/4 bridge Wheatstone bridge with a commercial metallic gauge. The tensile testing experiment is shown in Figure 7.

## 4. Results and Discussions

**Figure 8.**
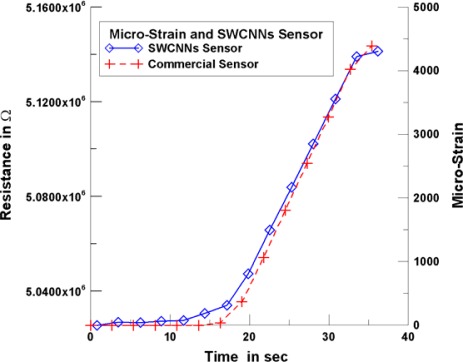
SWCNNs strain sensors and commercial strain sensors measurement.

In tensile testing, a commercial strain sensor was used to measure the strain of the test piece. Figure 8 shows the resistance variation and strain variation of a commercial strain sensor and the SWCNNs strain sensor (with growing time of 20 min, line width of 10 μm). Figure 8 shows that, within 0 to 15 s, no variation of resistance of commercial strain sensor occurred; however, a slight variation resistance of SWCNNs strain sensor was observed.

The tensile test specimens began to stretch within 15 to 33 s, and the resistance values of the commercial sensors and SWCNNs sensors increased. The strain curves of the commercial sensors and SWCNNs sensors began to overlap slowly when the stretch reached 1,800 microstrains. The noise did not have any interference effect on the SWCNNs strain sensors when the strain amount was sufficiently large. The curve began to produce non-linear phenomena when the stretch reached 4,000 microstrains, which indicates that the tensile test specimens began to produce plastic deformation. Therefore, the stretch test scope was limited to 3,500 microstrains in the later tests.

**Figure 9.**
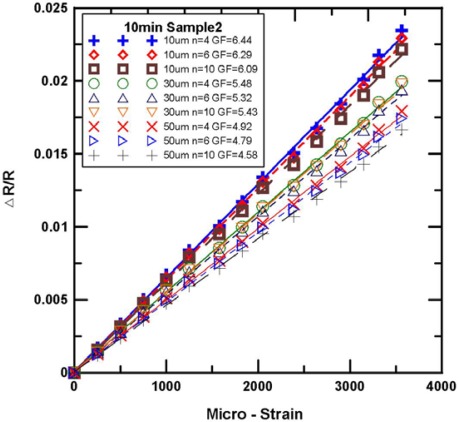
The strain sensitivities of the SWCNN strain sensor (growth time of 10 min, sample 2).

**Figure 10.**
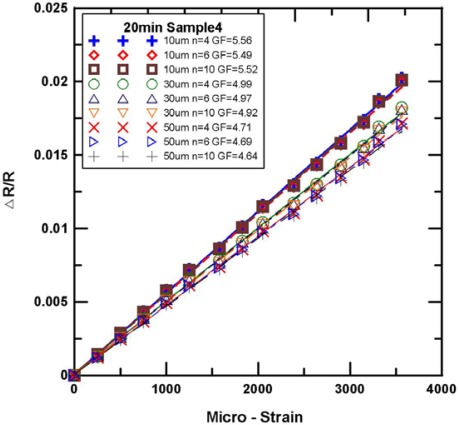
The strain sensitivities of the SWCNN strain sensor (growth time of 20 min, sample 4).

Figures 9 and 10 show the experimental results for the tensile test of the Parylene-based SWCNN strain sensors. Nine strain sensors with various lengths were obtained. The relative changes of the resistance of the strain sensors were increased when tension loading was applied. The gauge factor was calculated from the graph by the following equation:

(1) ΔR/R=G×ε

where ΔR/R is the relative change in resistance, and ε is the strain measured by the strain gauge. G is the gauge factor of the SWCNNs strain sensors. The average gauge factor of the SWCNN strain sensors was 5.05. The gauge factors were higher than those of the commercial metallic foil strain gauge (the GF of the metal foil gauge is 2.1).

Figure 9 shows the single-walled carbon nanonet (growth time of 10 min and Sample 2) with the strain sensitivities of the strain sensor in the tensile test. We obtained the gauge factor of the sensor by calculating the slope of the curve from Figure 9. As shown in Figure 9, the gauge factor of 10 μm is 6.09 to 6.44, that of 30 μm is 5.32 to 5.48, and that of 50 μm is 4.58 to 4.92. Similarly, Figure 10 shows the single-walled carbon nanonet (growth time of 20 min and Sample 4) with the strain sensitivities of the strain sensor in tensile test. As shown in Figure 10, the gauge factor of 10 μm is 5.49 to 5.56, that of 30 μm is 4.92 to 4.99, and that of 50 μm is 4.64 to 4.71. As shown in Figures 9 and 10, the sensors with the same width have measure curves that are closer to each other, which indicate the proximity of the gauge factor.

**Figure 11.**
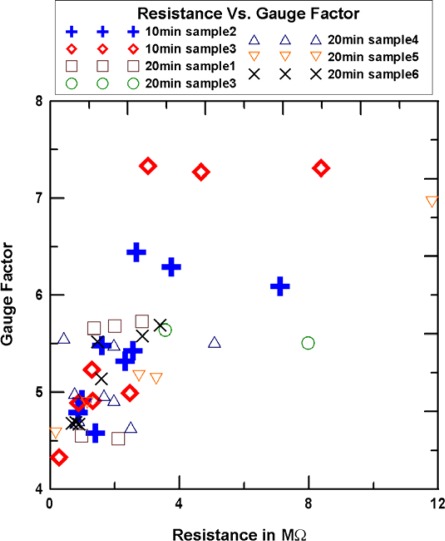
The relationship of resistance andgauge factor.

The relationship between gauge factors and resistances is shown in Figure 11. As seen in this figure, generally, the gauge factor of SWCNNs sensors with a growth time of 10 min is larger than that with a growth time of 20 min.

**Figure 12.**
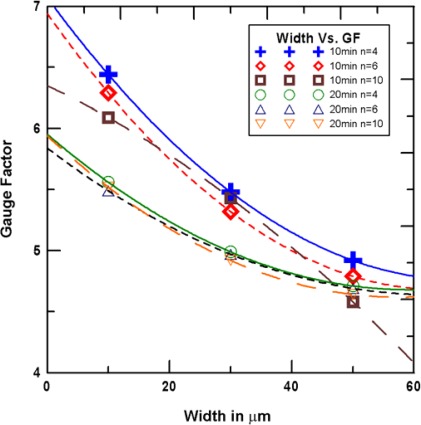
Therelationship of line width and gauge factor.

**Figure 13.**
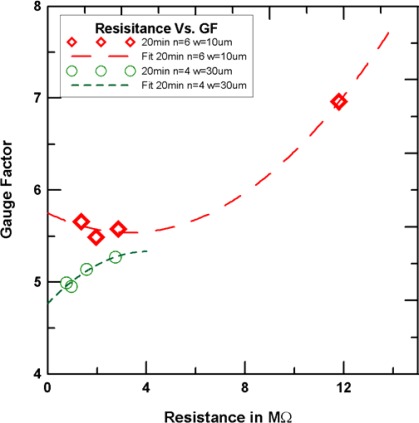
The relationship of resistance andgauge factor (with same line width and number of bar).

The line width effect on the gauge factor is shown in Figure 12. As shown, under the same number, the small line width has a large gauge factor. The difference in the number width and the gauge factor curve is smaller when the growth time is 20 min. This indicates that the higher the density of the single-walled carbon nanonet, the length change from the number has a smaller effect on the relation curve difference between line width and gauge factor. Figure 13 shows the relationship of resistance and gauge factor. As seen resistance and gauge factor are positively correlated. The electronic conductive path decrease as the network density decreases, resulting in an increase in resistance. Also, when the device is during a tension loading, the sparse network became more sparse, that resulting in an increase in gauge factor. Under the same line width and bar number, the large resistance indicates that the density of the single-walled carbon nanonet is low.

## 5. Conclusions

This study presents the design and fabrication of a strain sensor that uses single-walled carbon nanonets for strain sensing. The sensor was encapsulated between two layers of Parylene-C. These flexible strain sensors exhibited excellent strain sensitivities, and can be used to measure bone surface strain.
